# Inferring computational function of neuronal networks from multi-electrode array recordings: an evolutionary approach

**DOI:** 10.1186/1471-2202-12-S1-P167

**Published:** 2011-07-18

**Authors:** Thomas R Kiehl

**Affiliations:** 1 Nanobioscience Constellation, College of Nanoscale Science and Eng., University at Albany, Albany, NY, USA

## 

The availability of microelectrode array systems (MEA’s) has increased dramatically in recent years. Along with this increase in availability, these systems have also grown in capability. Modern systems stimulate and record *in-vitro* neuronal networks on an increasing number of channels. Closed-loop capabilities further expand the functionality of these systems. Future miniaturization of these systems and commensurate increased resolution promises a continued rise in the volume of data being produced in this domain.

It is difficult to determine if analysis tools are keeping pace with data generation. As of a few years ago Brown et al. saw a distinct need for investment in this vein[1]. It seems likely that data acquisition will continue to outstrip analysis just as it has in other bioinformatics domains. In 2006 Waganaar et al made available an extensive data set or recorded microelectrode array activity [2]. This data set contained both spontaneous and stimulated activity recorded regularly, from 58 unique cultures, of varying cell density, over a period of five weeks. While this study is referenced by hundreds of presenters and researchers, only a few groups have published analyses of this data. Patnaik et al stand out in that very small cohort as they sought to infer network structure from this data set based on the last 5 days of recordings from 6 cultures[3]. It is likely that these cultures were more static at this stage and were no longer developing connections at the same rate one may have observed in earlier time points.

**Figure 1:**
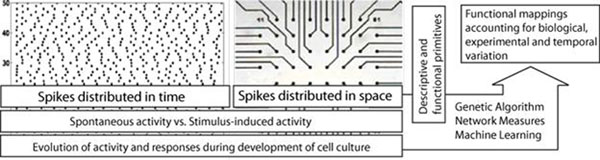
Generalized system view

Genetic algorithms (GA’s) and other evolutionary computing techniques have a proven track record in temporal knowledge discovery, network analysis, and machine learning[4][5]. In this work we demonstrate the feasibility of using a GA to tease out temporal and spatial relationships in the neuronal networks. Our analysis yields a functional and explanatory model of signal propagation on MEA’s and the development of neuronal networks *in-vitro*. As shown in figure 1 we seek to incorporate a broad range of MEA activity. The descriptive nature of the output from the evolutionary system can be tuned to include domain specific inferences. These models can present more actionable information for network design than their purely statistical counterparts.
